# Technical and scale efficiency of public community hospitals in Eritrea: an exploratory study

**DOI:** 10.1186/2191-1991-3-6

**Published:** 2013-03-16

**Authors:** Joses M Kirigia, Eyob Z Asbu

**Affiliations:** 1 World Health Organization, Regional Office for Africa, Brazzaville, Congo; 2 Department of Health Systems Financing, Health Authority, Abu Dhabi, United Arab Emirates

**Keywords:** Technical efficiency, Scale efficiency, Public hospitals, Data envelopment analysis

## Abstract

**Background:**

Eritrean gross national income of Int$610 per capita is lower than the average for Africa (Int$1620) and considerably lower than the global average (Int$6977). It is therefore imperative that the country’s resources, including those specifically allocated to the health sector, are put to optimal use. The objectives of this study were (a) to estimate the relative technical and scale efficiency of public secondary level community hospitals in Eritrea, based on data generated in 2007, (b) to estimate the magnitudes of output increases and/or input reductions that would have been required to make relatively inefficient hospitals more efficient, and (c) to estimate using Tobit regression analysis the impact of institutional and contextual/environmental variables on hospital inefficiencies.

**Methods:**

A two-stage Data Envelopment Analysis (DEA) method is used to estimate efficiency of hospitals and to explain the inefficiencies. In the first stage, the efficient frontier and the hospital-level efficiency scores are first estimated using DEA. In the second stage, the estimated DEA efficiency scores are regressed on some institutional and contextual/environmental variables using a Tobit model. In 2007 there were a total of 20 secondary public community hospitals in Eritrea, nineteen of which generated data that could be included in the study. The input and output data were obtained from the Ministry of Health (MOH) annual health service activity report of 2007. Since our study employs data that are five years old, the results are not meant to uncritically inform current decision-making processes, but rather to illustrate the potential value of such efficiency analyses.

**Results:**

The key findings were as follows: (i) the average constant returns to scale technical efficiency score was 90.3%; (ii) the average variable returns to scale technical efficiency score was 96.9%; and (iii) the average scale efficiency score was 93.3%. In 2007, the inefficient hospitals could have become more efficient by either increasing their outputs by 20,611 outpatient visits and 1,806 hospital discharges, or by transferring the excess 2.478 doctors (2.85%), 9.914 nurses and midwives (0.98%), 9.774 laboratory technicians (9.68%), and 195 beds (10.42%) to primary care facilities such as health centres, health stations, and maternal and child health clinics. In the Tobit regression analysis, the coefficient for OPDIPD (outpatient visits as a proportion of inpatient days) had a negative sign, and was statistically significant; and the coefficient for ALOS (average length of stay) had a positive sign, and was statistically significant at 5% level of significance.

**Conclusions:**

The findings from the first-stage analysis imply that 68% hospitals were variable returns to scale technically efficient; and only 42% hospitals achieved scale efficiency. On average, inefficient hospitals could have increased their outpatient visits by 5.05% and hospital discharges by 3.42% using the same resources. Our second-stage analysis shows that the ratio of outpatient visits to inpatient days and average length of inpatient stay are significantly correlated with hospital inefficiencies. This study shows that routinely collected hospital data in Eritrea can be used to identify relatively inefficient hospitals as well as the sources of their inefficiencies.

## Background

Eritrea is situated in the Horn of Africa. It is bordered by Sudan to the North and West, the Red Sea to the East, Ethiopia to the South and Djibouti to the Southeast [1]. The estimated population in 2009 was 5.073 million. The annual population growth rate of 3.6% was higher than the average for Africa (2.5%) and for the world (1.2%) [2]. Approximately 21% of the population lives in urban areas, compared to 38% and 50% for Africa and the world, respectively. Eritrea’s gross national income per capita stood at Int$610 in 2009 [2], which was lower than the Int$1620 reported for Africa, and considerably lower than the Int$6977 global average.

Eritrean health indicators are much better than those reported for the African Region as a whole. For example, life expectancy at birth of 66 years in 2009 was 12 years higher than the regional average and just two years lower than the global average. As shown in Additional file 1: Appendix, the infant mortality rate, under-five mortality rate, adult mortality rate and maternal mortality ratio for Eritrea were far better than the averages for Africa and the world [2]. The country’s HIV/AIDS, malaria, and tuberculosis cause-specific mortality rates are far much lower than the regional averages. For example, HIV/AIDS cause-specific mortality rate for Eritrea was five times lower than the regional average.

What kind of resources did Eritrea use to achieve these outcomes? In 2007 there were a total of 378 health facilities, including 26 hospitals (20 secondary hospitals and 6 national referral hospitals), 56 health centres, 2 health stations, 7 maternal and child health clinics (MCHC), and 107 clinics [3]. There were a total of 3909 beds, of which 63.3% were in hospitals, 29.1% in health centres and 1.6% in MCHC - 11.3 beds for every 10,000 people.

This health-care system was run by 210 medical doctors, 994 nurses, 1581 associate nurses, 48 pharmacists, 103 pharmacy technicians, 249 laboratory scientists, 67 radiology technicians, and 98 public health technicians (PHTs) [3]. To get a sense of what this means in regional and global terms Table 1 compares densities of four main categories of health workforce in Eritrea against the averages for the WHO African Region and the world [2,4], and shows that in every category Eritrea employs far fewer health workers per 10,000 of population than the region as a whole. There are, for example, five times fewer physicians per 10,000 of population than the regional average. The comparison is even more striking between Eritrean and global health workforce-to-population ratios [2]. Even though we compare average health workforce densities, we are cognizant that in both economically developed and developing countries, there are inequities in access to health facilities across wealth quintiles and place of resident (rural versus urban).

**Table 1:** Health workforce density in Eritrea compared to African Region and global averages in 2008

**Health workforce and infrastructure**	**Eritrea**	**WHO African Region**	**Global**
Physicians density (per 10 000 population)	0.5	2.3	14
Nursing and midwifery personnel density (per 10 000 population)	5.8	10.9	29.7
Dentistry personnel density (per 10 000 population)	<0.05	0.3	3
Pharmaceutical personnel density (per 10 000 population)	0.2	0.8	4.1
Environment and public health workers density (per 10 000 population)	0.2	0.4	…

Source: WHO [2].

It is widely accepted that improved efficiency is one of the four overarching goals of health systems [5,6]. There is a growing realization among health policy makers in the African Region of the need to utilize scarce health sector resources more efficiently [7], a good indication of which being the statement made by the 46 WHO Member States of the African Region in 2006 stressing their commitment to increasing the efficiency of health interventions and improving the allocation and management of health sector resources [8].

Since the adoption of the regional health financing strategy in 2006, a growing number of countries have undertaken health facility efficiency studies to guide the development of interventions to reduce waste of scarce health system resources. Since 2000 health facility efficiency studies have been undertaken in thirteen African countries, including Angola [9], Benin [10], Botswana [11], Burkina Faso [12], Ethiopia [13], Ghana [14,15], Kenya [16,17], Namibia [18], Nigeria [19], Seychelles [20], Sierra Leone [21,22], South Africa [23-25], and Zambia [26,27]. These studies demonstrate that DEA is an important tool for policy advice. To date, no health facility efficiency study has been conducted in Eritrea.

The study draws on Eritrean hospital data for 2007 to explore hospital efficiency at that time, and to demonstrate how an efficiency study could have informed decision-making. Three research questions are addressed: Were the public community hospitals in Eritrea relatively technically efficient in 2007? What were the magnitudes of output increases and/or input reductions needed for inefficient hospitals to operate relatively efficiently? How are efficiency scores for hospitals correlated with institutional and contextual/environmental variables?

The specific objectives of the study were: (a) to estimate the relative technical and scale efficiency of public secondary level community hospitals in Eritrea in 2007; (b) to estimate the magnitudes of output increases and/or input reductions that would have been required to make relatively inefficient hospitals more efficient; and (c) to estimate using Tobit regression analysis the impact of institutional and contextual/environmental variables on hospital inefficiencies.

## Methods

### Efficiency concepts

Skaggs and Carlson [28] define economic efficiency as obtaining the maximum benefit from a given cost or minimizing the cost of a given benefit. In other words economic efficiency means obtaining the maximum net gain (difference between the benefit received and the cost incurred) from an action. The authors further explain that economic efficiency comprises both technical efficiency (producing without waste) and allocative efficiency (allocating resources to their most high value uses).

In the context of health, allocative efficiency describes the use by a health facility or decision making unit (DMU) of health system inputs in the proportion that minimizes the cost of production, given input prices [29-31]. Estimation of allocative efficiency requires data on quantities of health service outputs, health system inputs, and input prices.

On the other hand, technical efficiency describes the production by a health DMU of the optimal/maximum quantity of outputs from the available health system inputs [29-31]. Alternatively, technical efficiency can be said to be achieved where a health decision making unit produces a given level of health service outputs with the least health system inputs, e.g. health workforce, pharmaceutical and non-pharmaceutical supplies, capital inputs (buildings, beds, equipment, vehicles), and community resources.

The technical efficiency of a health DMU can be broken down into pure technical efficiency and scale efficiency. Pure technical efficiency denotes health decision making unit technical efficiency that cannot be attributed to deviations from optimal scale (scale efficiency). Whereas scale efficiency is a measure of the extent to which a health decision making unit deviates from optimal scale (defined as the region in which there are constant returns to scale in the relationship between outputs and inputs) [30,31].

Salvatore [29] defines returns to scale as the extent to which health system output changes as a result of a change in the quantity of all health system inputs used in production. Where the quantity of all hospital inputs is increased by a given proportion, a constant return to scale is achieved when health service outputs increase in the same proportion. Thus, an increasing return to scale is achieved if output increases by a greater proportion than the increase in inputs and a decreasing return to scale is achieved where output increases by a smaller proportion than the increase in inputs.

**Figure 1:**
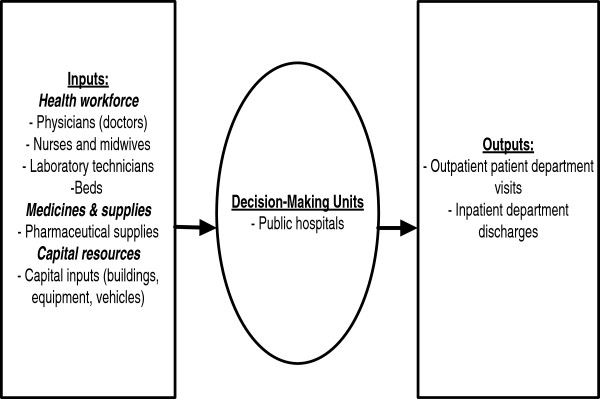
Eritrean hospital inputs, process and outputs.

Hospitals use multiple health system inputs to produce multiple health service outputs. Figure 1 depicts the relationship between health system inputs, the production process, and the outputs/results.

**Figure 2:**
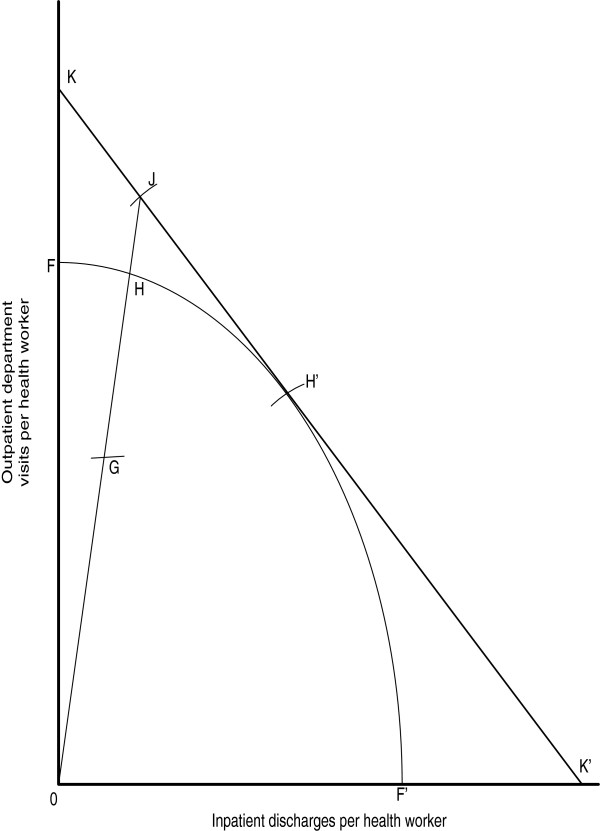
Farrell output-oriented efficiency measure.

Figure 2 was adapted from Coelli [32] and Farrell [33] to illustrate the Farrell output-orientated efficiency measure. Four hypothetical hospitals (G,H,H’,J) employs one health system input called ‘health worker’ to produce two hospital outputs, (i) outpatient department visits, and (ii) inpatient discharges. By dividing each output by the input we obtain the ratios that are on the y-axis and x-axis, i.e. outpatient department visits per health worker, and inpatient discharges per health worker. FF’ is the production possibilities frontier showing the upper limit of production possibilities. No hospital is capable of producing outputs beyond that frontier given its current health system inputs and technology endowment. Thus, any hospitals, such as H and H’, operating at the FF’ frontier are said to be relatively efficient, while any hospital, such as G, operating below that frontier is deemed relatively inefficient.

It is apparent that efficiency is never absolute; instead it is always assessed relative to some criterion. In their definition of relative efficiency, Cooper et al. [34] explain that “..a DMU is to be rated as fully (100%) efficient on the basis of available evidence if and only if the performances of other DMUs does not show that some of its inputs or outputs can be improved without worsening some of its other inputs or outputs (p.3).”

The output-orientated technical efficiency (TE) of hospital G is the ratio, *TE*_0_ = 0*G*/0*H*, which is the amount by which its health service outputs could be increased without recourse to extra inputs. According to Coelli [32], if health system input and output prices were available an isorevenue line, KK’, could be drawn and allocative efficiency (AE) defined as *AE*_0_ = *OH*/*OJ*, which indicates the reduction in production cost that would occur if production were to occur at the allocatively and technically efficient point H’. Even though H and H’ are technically 100% efficient, only H’ is producing its health service outputs at the lowest cost.

Overall economic efficiency (EE) of hospital G can be defined as follows: *EE*_0_ = (0*G*/0*J*) = (0*G*/0*H*) × (*OH*/*OJ*) = *TE*_0_ × *AE*_0_. AE, EE and TE scores are bounded by zero (totally inefficient) and one (totally relatively efficient). Since we did not have the data on input prices in the Eritrean study, we estimated only the TE and SE scores of hospitals. The estimation of TE and SE scores requires only output and input quantities.

In this paper a two-stage Data Envelopment Analysis (DEA) method was used to estimate efficiency of hospitals and to explain the inefficiencies.

### DEA analytical framework

Over the last half century efficiency has been calculated relative a frontier function using either non-parametric mathematical programming methods such as the Data Envelopment Analysis (DEA) or econometric/regression methods.

In the first stage, we use DEA to estimate the efficient frontier and the hospital-level efficiency scores. The main advantage of DEA is that it is able to deal with DMUs that employ multiple inputs to produce multiple outputs or services, which is typical of health systems units, e.g. hospitals and health centres. In addition, DEA not only identifies inefficient decision-making units but also permits analysis of sources of inefficiency and quantification of magnitudes of inefficiencies in the use of hospital inputs and production of outputs. It is for these reasons that, we considered DEA appropriate for the purposes of this study.

Following Charnes, Cooper and Rhodes (CCR) [35] the technical efficiency of a health decision making unit (a hospital) can be expressed as a maximum ratio of total sum of weighted outputs to total sum of weighted inputs. That is:

(1) $$\mathrm{Efficiency}=\left(\frac{\mathrm{Weighted}\;\mathrm{sum}\;\mathrm{of}\;\mathrm{hospital}\;\mathrm{outputs}}{\mathrm{Weighted}\;\mathrm{sum}\;\mathrm{of}\;\mathrm{hospital}\;\mathrm{inputs}}\right)\;$$

Assuming that there are *n* hospitals, each with *m* hospital inputs and *s* hospital outputs, the relative efficiency score of a given hospital *(TE*_*0*_*)* is obtained by solving the following output-orientated CCR linear programming model [35].

(2) $$\max TE_{0}\left(u,v\right)=\left(\frac{\sum_{r=1}^{s}\mu_{r}y_{r0}}{\sum_{i=1}^{m}v_{i}x_{i0}}\right)\;$$

$$\begin{array}{l} \mathrm{Subject}\;\mathrm{to}:\;\left(\frac{\sum_{r=1}^{s}\mu_{r}y_{\mathrm{rj}}}{\sum_{i=1}^{m}v_{i}x_{\mathrm{ij}}}\right)\leq 1;j=1,2,\ldots ,n\\ \;u_{i}\geq 0;i=1,2,\ldots ,m;\\ \;v_{r}\geq 0;r=1,2,\ldots ,s; \end{array}$$

Where:

*TE*_*0*_= the efficiency score of hospital 0;

*x*_*ij*_= the amount of health system input *i* utilized by the *j*^*th*^ hospital;

*y*_*rj*_= the amount of health system output *r* produced by the *j*^*th*^ hospital;

*u*_*i*_ = weight given to health system input *i*;

*v*_*r*_ = weight given to output *r*

If the denominator ($\sum_{i=1}^{m}u_{i}x_{i0}=1$) of equation (2) of the hospital is set equal to one, the transformed linear programming model for hospital 0 can be written as follows:

(3) $$\begin{array}{l} \max TE_{0}=\sum_{r=1}^{s}v_{r}y_{r0}\;\\ \;\mathrm{subject}\;\mathrm{to}:\sum_{r=1}^{s}v_{r}y_{\mathrm{rj}}-\sum_{i=1}^{m}u_{i}x_{\mathrm{ij}}\leq 0;j=1,2,\ldots ,n\\ \sum_{i=1}^{m}u_{i}x_{i0}=1\\ u_{i}\geq 0;i=1,2,\ldots ,m\\ v_{r}\geq 0;r=1,2,\ldots ,s \end{array}$$

The CCR model assumes constant returns to scale, meaning that all observed production combinations can be scaled up or down proportionally, i.e. not allowing for economies or diseconomies of scale. In other words, the model assumes that DMUs are able to linearly scale the inputs and outputs without increasing or decreasing efficiency [35]. CCR precludes the existence of variable returns to scale, where variable returns to scale denotes the changes in hospital outputs as hospital inputs change by the same proportion.

Suppose a hospital increases all its inputs by the same proportion. There are three possible scenarios: (i) its output(s) increases in line with the increase in inputs, which implies that there are constant returns to scale; (ii) its output(s) increases more than the increase in inputs, implying increasing returns to scale; or (iii) its output(s) increases less than the increase in inputs, which implies decreasing returns to scale [36].

In reality, a hospital can manifest constant returns to scale, increasing returns to scale or decreasing returns to scale depending on whether it is experiencing economies of scale or diseconomies of scale. Constant returns to scale occur in a situation where economies of scale are exhausted, and where health system inputs (factors of production) are perfectly divisible. The presence of increasing returns to scale may indicate indivisibilities in certain hospital inputs (e.g. diagnostic equipment, operating theatre), and greater scope for health workforce specialization as the scale/size of production increases. On the other hand, decreasing returns to scale can result when the large scale of production leads to cumbersome lines of communication between top hospital management and the health workforce in departments and wards, which lead to a decrease in managerial efficiency. Decreasing returns to scale could also occur due to over-utilization of abilities and skills of an entrepreneur [36].

Therefore, the application of the CCR model where hospitals are not operating at an optimal scale yields technical efficiency scores that are contaminated by scale efficiencies. In order to circumvent this problem, Banker, Charnes and Cooper (BCC) [37] introduced a slight modification in the CCR model to come up with a BCC model that allows the estimation of pure technical efficiencies. It was for this reason that we estimated the following output-oriented variable returns to scale BCC model:

(4) $$\begin{array}{l} \max TE_{0}\left(\mu ,v\right)=\;\sum_{r=1}^{s}\mu_{r}y_{\mathrm{ro}}+u_{0}\;.\\ \mathrm{Subject}\;\mathrm{to}:\\ \sum_{i=1}^{m}v_{i}x_{\mathrm{ij}}=1\\ \sum_{r=1}^{s}\mu_{r}y_{\mathrm{ro}}-\sum_{i=1}^{m}v_{i}x_{\mathrm{ij}}+\;u_{0}\leq 0,j=1,2,\ldots ,n\\ \mu_{r}\geq \varepsilon ,r=1,2,\ldots ,s\\ v_{i}\geq \varepsilon ,\;i=1,2,\ldots ,m\\ u_{0}\;\mathrm{is}\;\mathrm{unconstrained}\;\mathrm{in}\;\mathrm{sign}. \end{array}$$

Where: ε is an infinitesimal non-Archimedean quantity greater than zero. A value of *u*_0_ > 0 implies increasing returns to scale; *u*_0_ < 0 means decreasing returns to scale; and *u*_0_ = 0 denotes constant returns to scale. Thus, the above BCC model permits both the separation of technical and scale efficiencies, and determination of whether individual hospital’s operations were in regions of increasing, constant or decreasing returns to scale.

The linear programming model (4) shown above was run 19 times (equal to sample size) in identifying the relative technical efficiency scores of all the hospitals in the sample. DEA by default assigns weights to each hospital’s inputs and outputs in a way that maximizes its technical efficiency score. A hospital is considered to be technically efficient if it scores one, implying 100% relative technical efficiency, whereas a score of less than one implies that it is relatively technically inefficient, compared to peers in its efficiency reference set.

The scale efficiency score for each hospitals was obtained by dividing the constant returns to scale technical efficiency score by the variable returns to scale technical efficiency score [31,32]. A scale efficiency score of one implies that the hospital in question is operating at optimal scale or size. If the scale efficiency score is less than one, then the hospital is either too small or too big relative to its optimal size.

### Output-orientation

According to Coelli [32], where DMUs are given a fixed quantity of resources (inputs) and asked to produce as much output as possible, an output orientation is more appropriate. In the Eritrean context, the staffing capacity of each public hospital is determined centrally by the Ministry of Health, and thus individual hospital managers do not have any control over the size of the health workforce, and therefore of their inputs. In addition, hospital managers have no control over the size of the hospitals they run. It was for this reason that we used output-orientated DEA.

### Data

In 2007 there were a total of 20 secondary level public community hospitals in operation, the majority of which maintained good medical records. During first contact with a hospital, a patient card is opened and kept in the medical records office, and then updated during subsequent visits. The hospital medical records clerk collates daily summaries of the numbers of preventive visits, outpatient curative visits, admissions and discharges by ward. Every month, hospitals send a summary of data on selected output and input indicators to the management information system housed at the Ministry of Health headquarters. Each year the health management information system office analyses the data from all hospitals and publishes an annual health service activity report.

All hospitals, with the exception of one that did not report data on outputs, were included in the study. Input and output data were obtained from the Eritrea Ministry of Health annual health service activity report of 2007 [3]. The Eritrean hospitals were assumed to employ four inputs to produce two health service outputs, as outlined below.

Ideally, the statistical variable used to measure labour input is the hours actually worked by category of health worker, rather than the numbers of persons employed. Relying on numbers of health workers obscures changes in average hours worked due to, for example, absence from work. Because this study is based on the analysis of secondary data, routinely collated by management information, we did not have information on quantities of hours actually worked. We therefore used headcounts of health workers as proxies of labour input.

In a hospital context, physical capital goods include building space, beds, medical (diagnostic and therapeutic) equipment, and vehicles (including ambulances). Those capital goods are repositories or warehouses of physical capital services (measured as total machine hours) that are the actual input in the hospital production process. Since information on capital services, measured in machine hours, is not routinely collected and archived by management information, we used the number of hospital beds as a proxy.

#### Inputs

Input 1: Number of physicians (doctors)

Input 2: Number of nurses and midwives

Input 3: Number of laboratory technicians

Input 4: Number of operational beds and cots

#### Outputs

Output 1: Number of outpatient department visits

Output 2: Number of inpatient department discharges

Therefore, the choice of the above-mentioned inputs and outputs was guided by three considerations, namely: past studies undertaken of hospitals in Africa, which also employed similar inputs and outputs [9-27]; the availability of relevant data in the ministry of health annual health service activity report for 2007 [3]; and the availability of data that is routinely compiled by hospitals. Regarding the latter, we wanted to demonstrate ways in which the Eritrean ministry of health can get added informational value from such data without investing extra resources. The inputs and outputs data were used as reported in the ministry of health annual health service report without any processing or manipulation.

The inputs and outputs data were entered into computer using Excel software. The technical and scale efficiency scores for the hospitals were computed using DEAP 2.1 programme developed by Professor Tim Coelli [32].

### Explaining inefficiency through Tobit regression analysis

In the second stage, the DEA efficiency scores computed in the previous section were regressed against some institutional factors which are at the discretion of the hospital management and selected contextual/environmental (non-discretionary) factors that are beyond their control to estimate their impacts on efficiency. The literature indicates that some of the factors that impact health facility efficiency include, for example, catchment population, distance, location (urban/rural), ownership (profit/not-for-profit), teaching status, payment source (out-of-pocket/health insurance), occupancy rate, average length of stay, outpatient visits as a proportion of inpatient days, and quality [25,38,39].

A variety of regression techniques have been applied in the second stage to estimate the impact of contextual factors on efficiency, including the ordinary least squares (OLS) and the maximum likelihood (ML) based probit, logit, and truncated regression (Tobit). A debate has been raging between two schools of thought over the statistical properties of the two-stage DEA estimator. In one school of thought, scholars such as Simar and Wilson [40] argue that because DEA output scores are biased and contextual/environmental variables are correlated to output and input variables, the conventional statistical inferences are invalid in the second-stage regression, and recommend use of bootstrap methods. In another school of thought, scholars such as Ramalho et al. [41], McDonald [42] and Ruggiero [43] have argued that econometric models such as probit, logit, and truncated regression (Tobit) can be used for second-stage estimation of the impact of contextual/environmental variables on efficiency.

Afonso and Aubyn [44] argue that “Even if Tobit results are possibly biased, it is not clear that bootstrap estimates are necessarily more reliable, based on a set of assumptions concerning the data generation process and the perturbation term distribution that may be distributed (p.1429).” In their empirical study, the censored normal Tobit results and bootstrap algorithms yielded very similar results. We estimated the Tobit model (or censored normal regression model) because DEA efficiency estimates are bounded between 0 and 1.

In the Tobit model, for computational convenience it is preferable to assume a censoring point at zero [45].

Following Asbu [46], the CRS DEA efficiency scores are transformed into inefficiency scores, left-censored at zero using the formula:

$$\mathrm{Inefficiency}\;\mathrm{score}=\left(\frac{1}{\mathrm{DEA}\;\mathrm{TE}\;\mathrm{score}}\right)-1$$

The Tobit model is formulated as follows [47,48]:

(5) $$\begin{array}{l} y^{*}=\beta_{i}x_{i}+\varepsilon_{i}\;\\ y_{i}=y_{i}^{*}\;\mathrm{if}\;y_{i}^{*}>0\\ y_{i}=0\;\mathrm{if}\;y_{i}^{*}\leq \;0\\ \;i=1,2,\ldots ,N \end{array}$$

Where: N is the number of observations; *y*_*i*_ is the observed inefficiency score, i.e. dependent variable; y^*^ is the latent dependent variable; *βi* is the *kx1* vector of unknown parameters; *x*_*1*_ is the *kx1* vector of explanatory/independent variables; and *ε*_*i*_ is an independently distributed error term assumed to be normal with zero mean and constant variance σ^2^.

Some relevant institutional and operating environment variables were omitted due to the dearth of data. Therefore, the estimated empirical model was:

(6) $$\begin{array}{l} \mathrm{Ineff}=\alpha +\beta_{1}\;\mathrm{OPDIPD}+\beta_{2}\mathrm{ALOS}+\beta_{3}\mathrm{POP}\\ \;+\beta_{4}\;\mathrm{REGION}+\varepsilon_{i} \end{array}$$

Where: INEFF is the inefficiency score; OPDIPD is the outpatient visits as a proportion of inpatient days; POP is the region population dichotomous dummy variable = 1 if region or zoba population is one million and above (Maakel, Gash-Barkam and Debub), 0 if less than one million (Anseba, Semienawi Keyih Bahri, and Debubawi Keyih Bahri); REGION is the region dichotomous variable = 1 if the hospital is situated in a highland region (Anseba, Debub, Maekel), 0 if the hospital is situated in a lowland region (Gashbarka, Semienawi Keyih Bahri, and Debubawi Keyih Bahri); α is the intercept term; *β*_*1*_ is the vector of unknown parameters or coefficients; and *ε*_*1*_ is the stochastic/random error term.

Based on past two-stage hospital efficiency studies [25], we would expect a positive relationship between hospital inefficiency (*Ineff*) and ALOS, POP and REGION. Thus, regression coefficients *β*_*2*_, *β*_*3*_ and *β*_*4*_are expected to assume a positive sign. We would expect a negative relationship between the *Ineff* and *OPDIPD*, and thus, *β*_*1*_ should *a priori* assume a negative sign. Tobit coefficients indicate how a one unit change in an independent variable *x*_*i*_ alters the latent dependent variable y^*^.

By estimating equation 6, we wish to test two hypotheses. First, in order to test the overall significance of the equation, we state the joint null hypothesis as *H*_0_ : *β*_1_ =*β*_2_ = *β*_3_ = *β*_4_ = 0  and the alternative hypothesis *H*_*A*_ : *β*_1_ =*β*_2_ = *β*_3_ = *β*_4_ ≠ 0 . The joint null hypothesis is tested using the likelihood ratio test (LL).

Second, *β*_*n*_ is not significantly different from zero in either direction. Thus, the null (*H*_*0*_) and alternative hypotheses (*H*_*A*_) are: *H*_0_ : *β*_*n*_ = 0 ; and *H*_*A*_ : *β*_*n*_ ≠ 0 . The individual null hypotheses are tested using the t-distribution test.

Model 6 was estimated using STATA 10 statistical software [48]. The OPDIPD and ALOS data were obtained from the Eritrea Ministry of Health annual health service activity report 2007 [3]. The data on POP and REGION was obtained from Tewoldebrhan [49].

## Results and discussion

Table 2 presents the descriptive statistics (sum, minimum, maximum, mean and standard deviation) for inputs and outputs of Eritrean secondary public hospitals. In 2007 the 19 hospitals received 407,903 outpatient department visits and discharged 52,760 inpatients. Those outputs were produced using a total of 87 doctors, 1,008 nurses and midwives, 101 laboratory technicians, and 1,871 hospital beds. There was wide variation in both output and inputs across the different hospitals. The outpatient department visits varied from a minimum of 1,033 (Denden) to a maximum of 91,520 (Halibet), and inpatient discharges ranged between 160 (Denden) and 8,719 (Keren) patients. In terms of inputs there was considerable variation: the number of doctors varying between 0 (Tio, Senafe, Edaga Hamus, Denden) and 33 (Halibet); nurses, midwives & nurse associates varying between 7 (Tio) and 192 (Halibet); laboratory technicians between 2 (Tio, Nakfa, Sanafe, Sembel) and 15 (Halibet); and hospital beds and cots between 12 (Edaga Hamus) and 276 (Mendefera).

**Table 2:** Descriptive statistics of the input and outputs for public hospitals (n=19)

	**Sum**	**Minimum**	**Maximum**	**Mean**	**Standard deviation**
**Outputs:**					
Number of outpatient department visits	407,903	1,033	91,520	21,469	19,857
Number of discharges	52,760	160	8,719	2,777	2,260
**Inputs:**					
Number of doctors	87	0	33	5	7
Number of nurses, midwives & nurse associates	1,008	7	192	53	42
Number of laboratory technicians	101	2	15	5	4
Number of beds and cots	1,871	12	276	98	68

### Technical efficiency

Table 3 shows scores for constant returns to scale technical efficiency, variable returns to scale technical efficiency, scale efficiency, and returns to scale and the efficiency reference set. The latter refers to the group of hospitals against which DEA located the relatively inefficient hospitals and the magnitudes of inefficiency.

**Table 3:** Output oriented DEA efficiency scores for hospitals in Eritrea

**DMUs (Hospitals)**	**Efficiency scores**			**Returns to scale**	**Reference set (lambda weights)**
	**crste**	**vrste**	**scale**		
Tio Mini Ho.	1	1	1	crs	
Assab Ho.	0.722	0.741	0.974	irs	Afabet ( 0.316); Sembel (0.251); Aquardat (0.313); Edaga (0.057); Tio (0.063)
Massawa	0.777	0.888	0.875	drs	Aquardat (0.245); Sembel (0.016); Afabet (0.622); Halibet (0.117)
Ghinda	0.908	1	0.908	drs	
Afabet	1	1	1	crs	
Nakfa	0.975	1	0.975	irs	
Keren	1	1	1	crs	
Aqurdat	1	1	1	crs	
Tesenei	0.893	0.903	0.989	drs	Peer (Lambda weight): Keren (0.176); Aquardat (0.824)
Adikeyh	0.988	1	0.988	drs	
Adiquala MH	0.948	1	0.948	irs	
Dekemhare	0.924	0.933	0.99	irs	Adiquala (0.313); Aquardat (0.366); Edaga Hamus (0.183); Sembel (0.065); Keren (0.072)
Mendefera	0.821	0.982	0.836	drs	Sembel (0.293); Keren (0.640); Halibet (0.067)
Senafe MH	1	1	1	crs	
Edaga Hamus MH	1	1	1	crs	
Hazhaz	0.922	0.963	0.957	drs	Afabet (0.388); Aqurdat (0.134); Sembel (0.257); Halibet (0.221)
Sembel	1	1	1	crs	
Halibet	1	1	1	crs	
Denden	0.279	1	0.279	irs	
**Min**	**0.279**	**0.741**	**0.279**		
**Max**	**1**	**1**	**1**		
**Mean**	**0.903**	**0.969**	**0.933**		
**SD**	**0.172**	**0.065**	**0.165**		

Note: crste = technical efficiency from CRS DEA; vrste = technical efficiency from VRS DEA; scale = scale efficiency = crste/vrste.

Eight (42%) hospitals were constant return to scale technically efficient, and the remaining 11 (58%) were relatively inefficient. Among the latter, 6 hospitals had a constant return to scale technical efficiency score of 91-99%, 2 scored 81-90%, 2 scored 71-80%, and 1 scored less than 71%. The mean constant return to scale technical efficiency was 90.3%, with a standard deviation of 17.2%. The average constant return to scale technical efficiency score varied from a minimum of 27.9% in Denden hospital to a maximum of 100% at Tio, Afabet, Karen, Aquardat, Sanafe, Edaga Hamus, Sembel and Halibet hospitals. Out of the eight relatively constant return to scale technically efficient hospitals, three (Tio, Sanafe and Edaga Hamus), had no medical doctor on the staff, which obviously raises issues regarding quality of care.

Thirteen (68%) hospitals were variable returns to scale technically efficient, scoring 100%, and the remaining 6 (32%) hospitals were variable returns to scale technically inefficient. Three of the inefficient hospitals had variable returns to scale technical efficiency scores between 91 and 99%, two scored between 81% and 90%, and one scored below 81%. The overall sample average variable returns to scale technical efficiency score was 96.9% (standard deviation = 6.5%), meaning that inefficient hospitals could, on average, produce 3.1% more health service outputs using their current input endowment. Assab hospital had the lowest variable returns to scale technical efficiency score, at 74.1%. Surprisingly, four of the hospitals with no doctors were relatively variable returns to scale technically efficient. Once again, further investigation would have been necessary to establish the relative quality of services provided by those hospitals.

### Scale efficiency

Eight (42%) hospitals had a SE score of 100%, meaning they were at the optimal size for their particular input–output mix. The remaining 11 (58%) hospitals had scale efficiency scores of less than 100% and were thus deemed scale inefficient. Distribution was as follows: 1 hospital had a scale efficiency score of less than 31%; 2 hospitals had a scale efficiency score between 81-90%; and 8 hospitals had a scale efficiency of between 91-99%. The average scale efficiency score was 93.3% (standard deviation = 16.5%), meaning that on average, the scale inefficient hospitals could reduce their size by 6.7% without affecting their current output levels. The Ghinda, Nakfa, Adikeyh, Adiquala and Denden hospitals constant returns to scale technical inefficiency was fully attributed to scale inefficiencies.

The results revealed that increasing the quantity of all hospitals inputs by a given proportion would result in:

• Constant returns to scale in 8 (42%) hospitals, implying that their health service outputs would increase in the same proportion. This means that Tio, Afabet, Keren, Aquardat, Sanafe, Edaga Hamus, Sembel and Halibet hospitals were operating at their most productive scale sizes.

• Increasing returns to scale in 5 (26%) hospitals, implying that their health service outputs would increase by a greater proportion. These hospitals (Assab, Nakfa, Adiquala, Dekemhare and Denden) thus needed to increase their size to achieve optimal scale, i.e. the scale at which there are constant returns to scale in the relationship between inputs and outputs.

• Decreasing returns to scale in 6 (32%) hospitals, implying that their health service outputs would increase by a smaller proportion. Therefore, Massawa, Ghinda, Tesenei, Adikeyh, Mendefera and Hazhaz hospitals would have needed to reduce their size to achieve optimal scale.

### Econometric analysis of the determinants of inefficiency

Table 4 presents the Tobit regression model results. The joint null hypothesis that *H*_0_ : *β*_1_ = *β*_2_ = *β*_3_ = *β*_4_ = 0  is rejected at the 5 percent level of significance because the computed Chi-square of 16.98 is greater than the critical Chi-square value of 9.49 for the four degrees of freedom. Therefore, we can conclude that the *H*_*A*_ : *β*_1_ = *β*_2_ = *β*_3_ =*β*_4_ ≠ 0 , i.e., the regression coefficients for the explanatory variables (*OPDIPD*,*ALOS*,*POP*,*REGION*) are not equal to zero.

**Table 4:** Results for Tobit model

**Variable**	**Coefficient**	**t-ratio**
OPDIPD	−1.333	−3.14
ALOS	0.152	3.91
POP	0.145	0.47
REGION	0.068	0.22
Constant	−0.249	−0.68
Sigma	0.412	
	8 left-censored observations at *Ineff ≥ 0*	
	11 uncensored observations	
Observations summary	0 right-censored observations	
Number of observations	19	
χ^2^(4)	16.98	
Prob > χ^2^	0.0019	
Pseudo *R*_*2*_	0.457	

Notes: The critical t-value for 15 degrees of freedom and a 5% two-tailed level of significance is 2.131 is less than computed t-ratios for OPDIPD and ALOS, so we can reject the null hypothesis of no effect in these cases and conclude that the two are statistically significant variables in explaining expected hospital inefficiency.

The coefficient for *OPDIPD* has a negative sign consistent with our *a priori* expectation, and is statistically significant at the 5 percent level of significance. A unit increase in the ratio of outpatient department visits to inpatient days would lead to a decrease in hospital expected inefficiency score by 1.333, holding all other variables in the model constant. The higher a hospital *OPDIPD*, the lower the predicted inefficiency score.

The coefficient for ALOS assumed a positive sign as expected, and was statistically significant at the 5 percent level of significance. If the ALOS increases by one day, hospitals’ expected inefficiency score would increase by 0.152 while holding all other explanatory variables constant. Thus, the higher a hospital’s ALOS, the higher the predicted inefficiency score.

The coefficients for POPULATION and REGION had a positive sign but were statistically insignificant. Therefore, population size of the region where a hospital is situated and the geographical landscape (highland or lowland) do not have a significant effect on the efficiency (inefficiency) level.

### Implications for policy

As illustrated in Table 5, DEA revealed Assab hospital to be relatively inefficient, scoring just 0.741 in terms of PTE. This means that in 2007 Assab hospital could have achieved its reported output levels using 25.9% less of each input. Assab hospital’s inefficiency was identified and measured by comparing it with its efficiency reference set (Afabet, Sembel, Aquardat, Edaga Hamus and Tio). In row ‘F’ of Table 5, we show that the weighting of the ERS hospitals’ inputs and outputs yields a hypothetical hospital (here called the composite hospital) that produces as much or more than Assab hospital, but also uses fewer inputs.

**Table 5:** Comparison of Assab hospital with its efficiency reference set hospitals

**Efficiency reference set hospitals**	**Outputs**		**Inputs**			
	**OPD visits**	**Discharges**	**Doctors**	**Nurses**	**Laboratory technicians**	**Beds**
**Afabet [A]**	13652^a^ x 0.316^b^ = 4314.032	742^a^ x 0.316^b^ = 234.472	1^a^ x 0.316^b^ = 0.316	17^a^ x 0.316^b^ = 5.372	3^a^ x 0.316^b^ = 0.948	47^a^ x 0.316^b^ = 14.852
**Sembel [B]**	39451^a^ x 0.251^b^ = 9902.201	2996^a^ x 0.251^b^ = 751.996	2^a^ x 0.251^b^ = 0.502	81^a^ x 0.251^b^ = 20.331	2^a^ x 0.251^b^ = 0.502	103^a^ x 0.251^b^ = 25.853
**Aquardat [C]**	17930^a^ x 0.313^b^ = 5612.09	4356^a^ x 0.313^b^ = 1363.428	6^a^ x 0.313^b^ = 1.878	31^a^ x 0.313^b^ = 9.703	4^a^ x 0.313^b^ = 1.252	78^a^ x 0.313^b^ = 24.414
**Edaga Hamus [D]**	13307^a^ x 0.057^b^ = 758.499	1075^a^ x 0.057^b^ = 61.275	0^a^ x 0.057^b^ = 0	73^a^ x 0.057^b^ = 4.161	3^a^ x 0.057^b^ = 0.171	12^a^ x 0.057^b^ = 0.684
**Tio [E]**	5681^a^ x 0.063^b^ = 357.903	650^a^ x 0.063^b^ = 40.95	0^a^ x 0.063^b^ = 0	7^a^ x 0.063^b^ = 0.441	2^a^ x 0.063^b^ = 0.126	35^a^ x 0.063^b^ = 2.205
**Composite hospital [F=A+B+C+D+E]**	20944.725	2452.121	2.696	40.008	2.999	68.008
**Assab [G]**	15518	1818	5	40	3	68
**Output increase or input reduction [H=F-G]**	5426.725	634.121	−2.304	0.008	−0.001	0.008
% Change [I=(H/G)*100]	34.97	34.88	−46.08	0.02	−0.03	0.01

Note: superscript ‘a’ = Actual output and input quantities; superscript ‘b’ = Lambda weights from DEA.

In this example, the composite hospital outputs and inputs are derived by multiplication of the DEA-generated weights of 0.316 (Afabet), 0.251 (Sembel), 0.313 (Aquardat), 0.057 (Edaga Hamus) and 0.063 (Tio) by those hospitals actual outputs and inputs. The composite hospital’s target (projected) number of outpatient department visits of 20944 is the sum of weighted outpatient department visits in Afabet (4314), Sembel (9902), Aquardat (5612), Edaga Hamus (758) and Tio (358).

The difference between the composite hospital’s outpatient department visits and Assab hospital’s outpatient department visits is 5427 (35%). Thus Assab hospital needs to increase output by 5427 outpatient department visits to match the composite hospital performance. Assab would also have to increase hospital discharges by 634 (35%) to become relatively technically efficient. Alternatively, it could achieve the same efficiency score by reducing the number of doctors by 2.3 (46%).

Table 6 shows the input reductions and/or output increases that would have been required to make the six variable returns to scale technically inefficient hospitals more efficient.

**Table 6:** Efficiency scores and actual and target inputs and outputs quantities for inefficient hospitals according to VRS assumption

**DMUs (Hospitals)**	**Score**	**Input/Output**	**Actual quantities**	**Target quantities**	**Difference**	**%**
Assab	0.741	Doctors	5	2.7	−2.30	−46.04
		Nurses, midwives & nurse associates	40	40.0	0.00	0.00
		Laboratory technicians	3	3.0	0.00	0.00
		Beds	68	68.0	0.00	0.00
		Outpatient department visits	15,518	20,938.7	5,420.7	34.93
		Inpatient department discharges	1,818	2,453.1	635.06	34.93
Massawa	0.888	Doctors	6	6.0	0.00	0.00
		Nurses, midwives & nurse associates	42	42.0	0.00	0.00
		Laboratory technicians	5	4.6	−0.36	−7.22
		Beds	137	74.4	−62.60	−45.70
		Outpatient department visits	21,542	24,255.2	2,713.2	12.59
		Inpatient department discharges	1,938	2,182.1	244.09	12.59
Tesenei	0.903	Doctors	6	5.8	−0.18	−2.93
		Nurses, midwives & nurse associates	40	40.0	0.00	0.00
		Laboratory technicians	6	5.2	−0.77	−12.75
		Beds	120	99.7	−20.29	−16.91
		Outpatient department visits	10,315	19,598.4	9,283.4	90.00
		Inpatient department discharges	4,627	5,125.9	498.94	10.78
Dekemhare	0.933	Doctors	3	3.0	0.00	0.00
		Nurses, midwives & nurse associates	45	45.0	0.00	0.00
		Laboratory technicians	5	4.2	−0.81	−16.24
		Beds	66	66.0	0.00	0.00
		Outpatient department visits	16,341	17,514.1	1,173.1	7.18
		Inpatient department discharges	2,863	3,068.5	205.53	7.18
Mendefera	0.982	Doctors	6	6.0	0.00	0.00
		Nurses, midwives & nurse associates	99	89.1	−9.91	−10.01
		Laboratory technicians	9	8.6	−0.37	−4.07
		Beds	276	172.8	−103.22	−37.40
		Outpatient department visits	34,595	35,219.0	623.97	1.80
		Inpatient department discharges	6,684	6,804.6	120.56	1.80
Hazhaz	0.963	Doctors	9	9.0	0.00	0.00
		Nurses, midwives & nurse associates	74	74.0	0.00	0.00
		Laboratory technicians	13	5.5	−7.47	−57.46
		Beds	110	101.1	−8.88	−8.07
		Outpatient department visits	36,669	38,065.8	1,396.8	3.81
		Inpatient department discharges	2,678	2,780.0	102.01	3.81

Health policy-makers have three broad strategies available to them for addressing inefficient resources use: (a) increasing coverage of health services; (b) reducing hospital inputs; and/or (c) organization/process changes in hospitals. Since the third strategy is beyond the scope of the current study we will focus on the first two strategies.

#### (a). Increasing coverage of health services

In order for the inefficient hospitals to have become relatively efficient, as a group, they would have needed to increase their outpatient department visits by 20611 (5.05%) and inpatient department discharges by 1806 (3.42%). Individually, to be relatively technically efficient, Assab hospital needed to increase its outpatient department visits and inpatient discharges by about 35%; Massawa hospital ought to have increased its outpatient department visits and inpatient discharges by 13%; Tesenei hospital should have increased its outpatient department visits and inpatient discharges by 90% and 11%, respectively; Dekemhare hospital ought to have increased its outpatient department visits and inpatient discharges by 7%; Mendefera hospital needed to have increased its outpatient department visits and inpatient discharges by 2%; and Hazhaz hospital should have increased its outpatient department visits and inpatient discharges by 4%.

In 2000 the United Nations General Assembly adopted the Millennium Declaration which contains eight Millennium Development Goals (MDG) [50]. Three of them are health goals: MDG4: reduce child mortality; MDG5: improve maternal health; and MDG6: combat HIV/AIDS, malaria and other diseases. As shown in Additional file 1: Appendix, coverage of some of the MDG4 health services such as vitamin A supplementation treatment for children with acute respiratory infections, and oral rehydration therapy for children with diarrhoea is low. The coverage of MDG5 health services such as antenatal care, skilled birth attendance, and postnatal care is also low, as is coverage of MDG6 health services such as antimalaria treatment for children under five with fever, insecticide treated nets, antiretroviral therapy for HIV-infected pregnant women, and treatment for tuberculosis, among others [2]. The regression analysis results reported earlier clearly indicate that an increase in the ratio of outpatient visits to inpatient days would have increased the efficiency of hospitals in Eritrea.

Additional file 1: Appendix shows that only 28% of births were attended by skilled birth attendants, and yet on the whole bed occupancy rates in the hospitals were very low. For example bed occupancy was just 32.1% in Assab hospital, and 28.5% in Mendedera hospital. Among the inefficient hospitals, the best score on bed occupancy was achieved by Hazhaz hospital, but even there, it was just over 50%. The fact that 72% of pregnant women gave birth without the assistance of skilled health personnel, amidst underutilized hospital bed capacity, implies that there were factors preventing those in need from accessing care. One of the likely barriers to access in Eritrea is out-of-pocket payments for health care. In 2009, household out-of-pocket spending on health accounted for 52% of the total expenditure on health in Eritrea [51]. Such high levels of out-of-pocket payments have been shown to correlate highly with the incidence of financial catastrophe and impoverishment.

As shown on Table 7, user fees at health facilities were a major component of out-of-pocket payment. In Eritrea the registration and consultation fees are set according to the level of the health facility and whether or not a patient has been referred from a lower health system level. One consultation payment entitles patient to free consultation for a period of one month regardless of the illness concerned, but as Asbu [52] indicates, in Eritrean hospitals, fees are charged for diagnostic tests, therapeutic procedures and drugs over and above the registration and consultation fees. Various exemptions are also in operation. For example, tuberculosis, leprosy, sexually transmitted diseases and mental illness patients are exempted. Children under five and pregnant women have exemption for preventive services only. Even though the fees shown on Table 7 might appear to be modest, they represent a serious barrier to health service access in a country where approximately half of the population lived below the national poverty line in 2006 [53]. It should also be noted that the majority of people, especially those residing in rural areas, also incur travel costs to reach health facilities, while losing income through workdays lost (productivity losses) [54].

**Table 7:** Level and structure of user fees in United States Dollars in 1995

**Item**	**Health station**	**Health centre**	**Sub-zone hospital**	**Zone hospital**	**Tertiary hospital**
Registration and consultation (non-referred)	0.476	0.794	1.587	1.746	2.540
Registration and consultation (referred)	-	-	0.794	0.952	1.111
Inpatient hotel charges per day (non-referred)	-	0.159	3.016	3.016	3.016
Inpatient hotel charges per day (referred)	-	-	1.508	1.508	1.508
Private wing hotel charges per day	-	-	-	5.238	5.238

Source: Asbu [52].

Note: The fees in local currency have been converted into US Dollars using the 1995 exchange rate of 1USD=6.3 Nakfa.

It is clear from the National Health Policy of 2010, that the Eritrea Ministry of Health is very much aware of the adverse effects of significant out-of-pocket payment. In strategic policy goal 7, the Government set out a plan to introduce a health-financing scheme that protects people from catastrophic expenditures and ensures sustainability of the system. The National Health Policy states that [55]:

“*The Government shall develop evidence based mechanisms of risk-sharing and cross subsidisation that facilitate solidarity and equity. This will be achieved through a phased introduction of health insurance scheme(s) that will be informed by actuarial and socio-economic feasibility studies. (pp.28-29).*”

In other words, the country plans to develop prepaid and pooled health financing to reduce over reliance on out-of-pocket payments to finance the national health system. The introduction of such prepayment mechanisms will most likely reduce the economic barriers to access to health care, and hence, contribute to improving the efficiency of hospitals [56].

#### (b). Reducing hospital inputs

As already noted the six hospitals that were variable returns to scale technically inefficient in 2007 could also have improved their relative efficiency by reducing their inputs by a total of 2.478 doctors (2.85%), 9.914 nurses and midwives (and associate nurses) (0.98%), 9.774 laboratory technicians (9.68%) and 194.995 beds (10.42%). The required input reductions for individual hospitals are set out in Table 6 where it is apparent that most hospitals would have benefited from reductions in the number of laboratory technicians and beds.

What could have been done with excess inputs in 2007? Given the national drive to attain the health Millennium Development Goals, it would not have been prudent to lay-off excess staff. The policy-makers could have considered reassigning excess health workers to health centres, health stations and maternal and child health clinics. They could also have explored the feasibility of creating mobile pools of excess health workers to go around health centres, health stations and maternal and child health clinics, providing outreach services. According to the United Nations Millennium Development Goals report 2011 [50], Eritrea is already on track to achieve Millennium Development Goals 4 and 5. The efficiency improvements identified in this paper would only have helped to accelerate what is already commendable progress in these areas.

On the other hand, excess beds could either have been transferred to primary health care facilities with bed shortages or sold to the non-governmental health sector. This would have ensured that the extra supply of beds would not have led to more admissions and longer stays, a phenomenon often referred to as Roemer’s Law [57].

### Limitations of the study

The study reported in this paper has a number of limitations. First, DEA is a deterministic technique. Thus, any deviation from the production possibilities frontier is attributed to inefficiency, whereas some of the deviations from the frontier may in fact be due to epidemics, civil war or natural disasters (e.g. flooding, earthquakes) leading to displacement of people.

Second, the analysis reported in this paper is based on hospital inputs and outputs data for 2007. Much has happened since 2007, notably in terms of the country’s socioeconomic and health development. It was for this reason that we stated at the outset that the results of this analysis are not meant to be uncritically fed into current decision-making, but rather to illustrate the potential usefulness of such efficiency analyses.

Third, due to the lack of data, this study did not include the expenditures on pharmaceuticals and non-pharmaceutical supplies among the inputs. Nor does the study take into consideration the differences that may exist between the categories of nurses and doctors in the various hospitals. In addition, even within the same health workforce category, the quality of labour input may vary depending on individual health worker skills, professional experience and health status.

Fourth, the hospitals included in the study were all secondary public community hospitals. However, because the study was based on secondary data, we had no way of knowing whether there might have been some variations in the severity of cases treated in each hospital. Significant differences in the severity of cases treated could affect the number of cases hospitals dealt with relative to their staff numbers and bed numbers, and could therefore have an impact on the results of the analysis. The hospitals treating a large number of severe cases, for example, may handle fewer cases, and will thus appear to be relatively inefficient.

### Suggestions for further research

a) There is a need for a Malmquist Total Factor Productivity Index analysis to measure the trends in efficiency and productivity of hospitals over time [9,11,20,30]. This would entail collecting inputs and outputs data for a number of years, e.g., from 2008 to 2012. Such an analysis would permit comparison of the current state of hospital efficiency with the situation prevailing in 2007.

b) There is a need for demand analyses studies to identify the significant determinants of households’ decisions to seek health care from hospitals, and for studies evaluating the effectiveness of various options for removing or reducing barriers to population access to essential health services [58].

c) In its 2010 National Health Policy, the Eritrean Ministry of Health sets out a plan to introduce hospital reforms (organizational changes) which include introducing hospital autonomy aimed at improving performance, quality of care, cost containment, and hence, sustainability of services. Autonomy usually entails a shift of financial and human resource management as well as service development planning responsibility from the Ministry of Health to the hospitals themselves. It is our hope that while developing the evidence-based Hospital Reforms Strategic Plan envisaged in the National Health Policy, the Ministry of Health will develop a set of indicators for tracking changes in efficiency and performance of hospitals over time. Monitoring and evaluation could begin with the above-mentioned Malmquist Total Factor Productivity Index analysis prior to implementation of hospital reforms in order to establish a meaningful baseline against which to measure progress.

## Conclusions

One of the strategic policy goals contained in Eritrea’s 2010 National Health Policy is to enhance efficiency, equity and quality of service delivery through health systems development.

The study met its objectives in (i) estimating the relative technical and scale efficiencies of 19 secondary public hospitals in Eritrea, (ii) in quantifying the magnitudes of output increases and/or input reductions that could have made inefficient hospitals more efficient in 2007, and (iii) in estimating the impact of institutional and contextual/environmental variables on hospital inefficiencies using Tobit regression analysis.

The findings from the first-stage analysis indicate that about 68% hospitals were variable returns to scale technically efficient. Only 42% hospitals achieved scale efficiency. The inefficient hospitals collectively could have become efficient by increasing their total outpatient visits and hospital discharges by 5.05%% and 3.42%. Therefore, in 2007 there was scope for treating an extra 20,611 outpatients and making 1,806 inpatient discharges per year with the existing hospital input endowments.

Alternatively, policy-makers might have considered boosting the quality of services provided by primary health care facilities by transferring the excess 2.478 doctors (2.85%), 9.914 nurses and midwives (0.98%), 9.774 laboratory technicians (9.68%), and 195 beds (10.42%) to health centres and maternal and child health clinics.

Our second-stage analysis show that the ratio of outpatient visits to inpatient days and average length of inpatient stay are significantly correlated to hospital inefficiencies. Thus, policy interventions that increase utilization of under-utilized hospital outpatient health services and reduce the hospital average length of stay would help to reduce inefficiencies. The population size of the region where a hospital is situated and the geographical landscape (highland or lowland) were not found to have a significant effect on hospital inefficiency level.

In a nutshell, even where inefficiencies existed within hospitals, it would **not** have been prudent to ameliorate them by down-scaling the health workforce. Instead, it would have been better to address such inefficiencies by either reassigning excess health workers to primary health care facilities or by increasing the coverage of health services offered in order to address the unmet need for hospital services.

## Competing interests

The authors declare that they have no competing interests.

## Authors’ contributions

JMK and EZA were equally involved in the literature review, data analysis, interpretation of the results, and drafting of the manuscript. Both authors read and approved the final manuscript.

## Supplementary Material

Additional file 1 **Appendix.** Comparison of Eritrea health statistics with those of the WHO African Region and the world. Click here for file
