# Supplementary material for: Technical and scale efficiency of public community hospitals in Eritrea: an exploratory study
Source: Health Econ Rev. 2013 Mar 16;3:6. doi: 10.1186/2191-1991-3-6 (PMC3605339; doi:10.1186/2191-1991-3-6)
Supplement: Additional file 1 — Appendix. Comparison of Eritrea health statistics with those of the WHO African Region and the world. [file 2191-1991-3-6-S1.docx]

Appendix: Comparison of Eritrea health statistics with those of the WHO African Region and the world

| **Life expectancy and mortality** | **Eritrea** | **WHO African Region** | **Global** |
| --- | --- | --- | --- |
| Life expectancy at birth a (years) | 66 | 54 | 68 |
| Infant mortality rate a (probability of dying by age 1 per 1000 live births) | 39 | 80 | 42 |
| Under-five mortality rate a (probability of dying by age 5 per 1000 live births) | 55 | 127 | 60 |
| Adult mortality rate a (probability of dying between 15 and 60 years per 1000 population) | 211 | 383 | 176 |
| Maternal mortality ratio (per 100 000 live births): 2008 | 280 | 620 | 260 |
| **Health service coverage** |  |  |  |
| At least 1 visit antenatal care coverage (%) | 70 | 74 | 80 |
| At least 4 visit antenatal care coverage (%) | 41 | 44 | 53 |
| Births attended by skilled health personnel (%) | 28 | 48 | 66 |
| Births by caesarean section a (%) | 2.7 | 3.6 | 14.8 |
| Neonates protected at birth against neonatal tetanus (%) | 86 | 82 | 83 |
| Measles immunization coverage among 1-year-olds (%) | 95 | 69 | 82 |
| DTP3 immunization coverage among 1-year-olds (%) | 99 | 71 | 82 |
| HepB3 immunization coverage among 1-year-olds (%) | 99 | 70 | 70 |
| Hib3 immunization coverage among 1-year-olds d (%) | 99 | 62 | … |
| Children aged 6–59 months who received vitamin A supplementation (%) | 38 | 45.9 | 38.9 |
| Children aged <5 years sleeping under insecticide-treated nets (%) | 4 | 17 | … |
| Children aged <5 years With fever who received treatment with any antimalarial | 4 | … | … |
| Children aged <5 years With ARI symptoms taken to a health facility | 43.6 | 43.9 | 77.2 |
| Children aged <5 years With diarrhoea receiving ORT (ORS and/or RHF) | 55.7 | 41 | 64.1 |
| Unmet need for family planning (%) | 27 | 24.8 | 11.2 |
| Contraceptive prevalence (%) | 8 | 24.4 | 62.7 |
| 2009 Antiretroviral therapy coverage (%) among HIV-infected pregnant women for PMTCT | 34 | 54 | 53 |
| 2009 Antiretroviral therapy coverage (%) among people with advanced HIV infection | 37 | 37 | 36 |
| 2000 Case-detection rate for all forms of tuberculosis (%) | 210 | 38 | 45 |
| 2009 Case-detection rate for all forms of tuberculosis (%) | 58 | 49 | 62 |
| 2000 Smear-positive tuberculosis treatment-success rate (%) | 76 | 71 | 69 |
| 2008 Smear-positive tuberculosis treatment-success rate (%) | 76 | 80 | 86 |
| **Health workforce and infrastructure** | **Eritrea** | **WHO African Region** | **Global** |
| Physicians | 215 | 173677 | 9171877 |
| Physicians density (per 10 000 population) | 0.5 | 2.3 | 14 |
| Nursing and midwifery personnel | 2505 | 805575 | 19379771 |
| Nursing and midwifery personnel density (per 10 000 population) | 5.8 | 10.9 | 29.7 |
| Dentistry personnel | 16 | 25772 | 1932650 |
| Dentistry personnel density (per 10 000 population) | <0.05 | 0.3 | 3 |
| Pharmaceutical personnel | 107 | 56198 | 2587043 |
| Pharmaceutical personnel density (per 10 000 population) | 0.2 | 0.8 | 4.1 |
| Environment and public health workers | 88 | 28657 | … |
| Environment and public health workers density (per 10 000 population) | 0.2 | 0.4 | … |
| Hospital beds (per 10 000 population) | 12 | 9 | 29 |
| Radiotherapy units (per 1 000 000 population) | 0 | 0.1 | 1.8 |

Source: WHO [2].
